# May the midline lumbar interbody fusion (MIDLIF) prevent the early radiographic adjacent segment degeneration? A minimum 3-year follow-up comparative study of MIDLIF in L4/5 with cortical bone trajectory screw versus traditional pedicle screw fixation

**DOI:** 10.1186/s12891-022-05363-0

**Published:** 2022-05-20

**Authors:** Bo Han, Hongtao Ding, Yong Hai, Yuzeng Liu, Li Guan, Aixing Pan, Xinuo Zhang, Peng Yin

**Affiliations:** Department of Orthopedics, Beijing Chao-Yang Hospital, Capital Medical University, GongTiNanLu 8#, Chao-Yang District, Beijing, 100020 China

**Keywords:** Midline lumbar interbody fusion, Cortical bone trajectory screw, Pedicle screw, Radiographic Adjacent segment degeneration, Sagittal alignment

## Abstract

**Study design:**

Retrospective cohort study.

**Objective:**

To compare the early radiographic adjacent segment degeneration (R-ASD) and regional lumbar sagittal alignment after midline lumbar interbody fusion (MIDLIF) with cortical bone trajectory (CBT) screw fixation (CBT-MIDLIF) and posterior lumbar interbody fusion (PLIF) with the traditional pedicle screw fixation (PS-PLIF) during long-term follow-up.

**Methods:**

All patients who underwent CBT-MIDLIF or PS-PLIF were identified by a retrospective consecutive case review. Radiographic parameters in cephalad adjacent segment (L3/4), including intervertebral space height (ISH), foraminal height (FH), foraminal width (FW), range of motion were assessed. Lumbar lordosis (LL), sacral slope (SS), L4–L5 Cobb angle, Cobb angle of the intervertebral space at L4–L5, and height of the anterior and posterior edges of the intervertebral space at L4–L5, were measured and compared on preoperative, postoperative, and 3-year follow-up radiographic evaluation.

**Results:**

Seventy-four patients underwent CBT-MIDLIF (CBT-MIDLIF group) and 114 patients underwent conventional PS-PLIF (PS-PLIF group). ISH, FH and FW were significantly smaller at 6-month follow-up than before operation with PS-PLIF (*p* < 0.001) but showed no significant changes with CBT-MIDLIF (*p* > 0.05). At the last follow-up, the changes in cephalad R-ASD parameters were more remarkable after PS-PLIF than after CBT-MIDLIF (*p* < 0.01). LL and SS were significant larger at the last follow-up than before operation in both groups (*p* < 0.001). Regarding long-term outcomes, the symptoms caused by degenerative spinal disorders significantly improved in both groups (*p* < 0.01).

**Conclusion:**

CBT-MIDLIF had less radiographic degeneration in the adjacent segment than PS-PLIF at 3-year follow-up. The lumbar sagittal alignment could be improved significantly and the surgical outcomes were satisfactory after either CBT-MIDLIF or PS-PLIF.

## Introduction

With the growing elderly population and improvement of medicine, the prevalence rates of osteoporosis and osteopenia with low back pain have increased markedly [1], resulting in a substantial economic and social burden on patients and the health-care system [2, 3]. Posterior lumbar interbody fusion (PLIF), which was first described by Cloward in 1953, has been indicated for patients with neurological claudication and segmental spinal instability with good surgical outcomes [4, 5]. However, midline lumbar interbody fusion (MIDLIF), which is a relatively new method for degenerative pathologies consisting of a posterior midline approach, microsurgical laminectomy, and CBT screw fixation, was presented as a valid alternative to the more traditional pedicle screw (PS) trajectory by Mizuno in 2014 [6].

Nevertheless, the regional lumbar sagittal alignment plays a crucial role in the pathogenesis of adjacent segment degeneration (ASD) and postoperative outcomes that should not be ignored. The PS has been widely used, but it is always associated with complications such as screw loosening due to osteoporosis, which leads to lumbar sagittal imbalance postoperatively [7]. In 2009, the cortical bone trajectory (CBT) was presented by Santoni et al. [8] as an alternative method to improve the holding screw strength and minimize loosening, as obtaining a stable fixation in patients with osteoporosis is difficult. Previous studies found superior biomechanical evaluations and surgical outcomes with CBT screw fixation than with the traditional PS fixation [9, 10]. Therefore, CBT has gradually been widely used in surgery in recent years.

However, long-term follow-up studies on radiographic ASD (R-ASD) and lumbar lordosis (LL) restoration of the CBT are currently lacking. The purpose of this study was to investigate the early R-ASD and regional lumbar sagittal alignment after MIDLIF with CBT screw fixation (CBT-MIDLIF) and compare them with those after PLIF with the traditional pedicle screw fixation (PS-PLIF) and to provide the basic guidelines for the clinical application of CBT-MIDLIF from long-term follow-up.

## Materials and methods

A retrospective consecutive case review was performed to identify all patients with degenerative spinal disorders who underwent CBT-MIDLIF or PS-PLIF between December 2015 and December 2018 at our institution. The inclusion criteria were as follows: (1) severe symptoms because of degenerative lumbar spine disorder such as lumbar spinal stenosis (LSS) or degenerative lumbar disc herniation (LDH); (2) with lumbar instability or significant lumbar disc degeneration where the Pfirrmann classification was above grade III [11]; (3) concomitant osteoporosis or osteopenia; (4) underwent CBT-MIDLIF or PS-PLIF at L4/L5; (5) at least 36 months of postoperative follow-up; and (6) complete radiological and clinical evaluations, including lumbar radiography and magnetic resonance imaging (MRI), after 1, 6 months and 3 years. The exclusion criteria were as follows: (1) concomitant spinal deformity, spinal tumor, spinal infection, or spinal trauma; (2) history of lumbar surgery; (3) no-mechanical complications complication during the perioperative period such as cardiopulmonary failure and anesthesia complications; and (4) insufficient follow-up time and incomplete or blurry anterior–posterior radiograph. Patients self-selected CBT-MIDLIF or PS-PLIF during preoperative interviews, and baseline data were collected and compared. This study was approved by our institutional review board, and informed consent was waived because of the retrospective study design, which was approved by the appropriate ethics review board (No.2019-KE-264).

### Surgical procedure

According to the preoperative informed consent, the surgeon fully communicated and introduced the operation and procedure of CBT-MIDLIF or PS-PLIF. All patients made their own choice of which surgical procedure during the preoperative conversation. The specified group of same surgeons performed the operation separately. In both groups, the same fluoroscopy was used to help the team locate the surgical site and maneuver the instrument accurately. All the patients in both groups underwent the decompression of the spinal stenosis decompression and interbody fusion, for which the PLIF technique was used with allogenous cancellous bone grafts put into the interbody space. Finally, the wound was closed in a standard fashion, typically with drainage on one side. All the patients were required to wear a thoracolumbosacral brace for 6 months.

#### Surgical technique of CBT-MIDLIF

CBT-MIDLIF was performed by three surgeons while paying more attention to the less invasiveness of the CBT screws, which were produced with the CD Horizon Solera Spinal System (Medtronic, Memphis TN, USA) with a 5.5-mm diameter and lengths ranging from 30 to 40 mm. Starting with a midline incision, a bilateral paraspinal muscle dissection was performed on the lateral portion of the facet joint with a self-retaining lumbar retractor system. In accordance with the technique described in detail in the previous literature, [8–10] the surgeons identified the starting point for CBT on the pars interarticularis. Through the pedicle, the CBT was prepared from the inferior to the superior direction and from the medial to the lateral direction, under the protection of the supra-adjacent segment.

#### Surgical technique of PS-PLIF

PS-PLIF was performed by only three surgeons while attaching more importance to the greater fixation strength of the PS-rod constructs, which were produced with the CD Horizon Legacy Spinal System (Medtronic, Memphis TN, USA) with a 6.5-mm diameter and lengths ranging from 40 to 50 mm. Starting with a midline incision, a bilateral paraspinal muscle dissection was performed to the tips of the transverse processes with the typical retractors moved and adjusted by the surgeons. Through the pedicle, a traditional PS was used to prepare the screw paths from the lateral to medial direction.

### Radiological and clinical evaluations

Demographic and clinical data were collected and anonymously handled by two researchers who were blinded to the study design. We adopted the cephalad R-ASD parameters that can better react to changes at L3–L4 to observe R-ASD rather than symptomatic ASD (S-ASD) at an early stage due to the limitation of follow-up time and the better observation of trend.

#### Radiographic evaluation

**Fig. 1:**
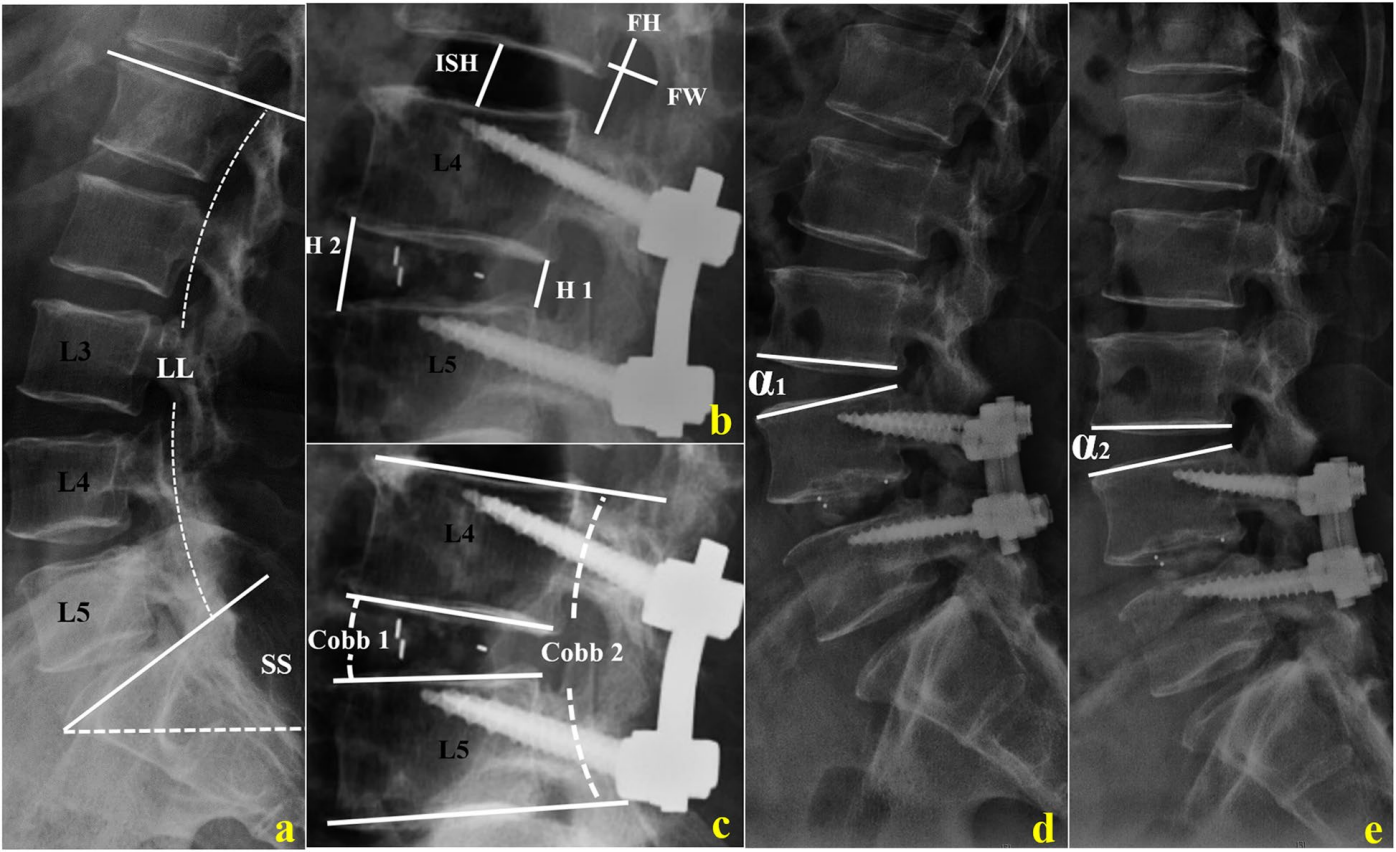
The measurements of lumbar lordosis (LL), sacral slope (SS) (**a**), intervertebral space height (ISH), foraminal height (FH), foraminal width (FW), the height of the anterior and posterior edges of the intervertebral space at L4–L5 (**b**), L4–L5 Cobb angle, and the Cobb angle of the intervertebral space at L4–L5 (**c**). Range of motion (ROM) is the difference between the angle (*α*_1_) at extension (**d**) and the angle (*α*_2_) at flexion (**e**). Cobb 1: the Cobb angle of the intervertebral space at L4–L5; Cobb 2: L4–L5 Cobb angle; H1: height of the posterior edges of the intervertebral space at L4–L5; H2: height of the anterior edges of the intervertebral space at L4–L5

The picture archiving and communication system was used for the radiographic measurements. On the preoperative and postoperative spinal radiographs, the radiographic parameters were measured and compared, including the intervertebral space height (ISH), foraminal height (FH), foraminal width (FW), range of motion (ROM) in cephalad adjacent segment (L3/4). LL, sacral slope (SS), L4–L5 Cobb angle, the Cobb angle of the intervertebral space at L4–L5, and the height of the anterior and posterior edges of the intervertebral space at L4–L5 (named H1and H2 separately) were measured and compared (Fig. 1). A 3-year postoperative MRI scan was used to evaluate the L3/4 spinal disc according to the lumbar intervertebral disc degeneration classification proposed by Pfirrmann et al. [11].

Grade I: The structure of the disc is homogeneous, with a bright hyperintense white signal intensity and a normal disc height. Grade II: The structure of the disc is inhomogeneous, with a hyperintense white signal; The distinction between nucleus and anulus is clear, and the disc height is normal, with or without horizontal gray bands. Grade III: The structure of the disc is inhomogeneous, with an intermediate gray signal intensity; The distinction between nucleus and anulus is unclear, and the disc height is normal or slightly decreased. Grade IV: The structure of the disc is inhomogeneous, with an hypointense dark gray signal intensity; The distinction between nucleus and anulus is lost, and the disc height is normal or moderately decreased. Grade V: The structure of the disc is inhomogeneous, with a hypointense black signal intensity; The distinction between nucleus and anulus is lost, and the disc space is collapsed.

#### Clinical outcome measurements

The clinical outcome was assessed by the Oswestry disability index (ODI), visual analog scale (VAS), and Japanese Orthopedic Association (JOA) scores to evaluate back pain, leg pain, and loss of function at pre-operation, post-operation and every follow-up. The patient's outpatient medical records were collected to investigate the presence of surgically related symptoms at the 1-month, 6-month, and last follow-up. The symptoms that were related to S-ASD of L3/4 such as back pain, leg pain, and numbness were documented in detail.

### Statistical analyses

All the statistical analyses were performed using SPSS Statistics Version 20.0 (IBM, Armonk, New York). Paired or independent *t* test and Mann–Whitney *U* test were used to analyze continuous data, and *χ*^2^ test and Fisher exact probability test for the enumeration data. A *p* value < 0.05 was considered statistically significant, and the *p* value < 0.0oa was recorded as 0.00.

## Results

According to the inclusion and exclusion criteria, there were 74 patients underwent CBT-MIDLIF (CBT-MIDLIF group) which were composed of 30 men and 44 women, with a mean age of 67. 6 years (range, 56–78 years). The mean follow-up time was 39.23 months (range, 36–43 months). A total of 114 patients underwent PS-PLIF (PS-PLIF group), including 57 men and 57 women, with a mean age of 62. 7 years (range, 51–75 years). The mean follow-up time was 43.23 months (range, 36–51 months). Details of the demographic and surgical characteristics of the patients in the two groups are listed in Table 1. We found no statistically significant differences between the two groups in terms of sex distribution, age, follow-up time, body mass index (BMI), bone mineral density of the lumbar spine, diagnosis distribution, number of interbody cages, and type of bone graft (*p* > 0.05). Meanwhile, there were 13 patients (17.57%) in CBT-MIDLIF group and 19 patients (16.6%) PS-PLIF suffered in S-ASD with the responsibility of L3/4 at last follow-up (*p* = 0.87).

**Table 1:** Summary of demographic parameters and surgical parameters

Variable	CBT Group *N* = 74	PS Group *N* = 114	*p* Value
Age (year)	67. 6 ± 9.8	62.7 ± 10.9	0.53
Sex(male/female)	30/44	57/57	0.23
BMI	27.52 ± 3.21	27.79 ± 5.56	0.71
Follow-up time(month)	39.23 ± 3.03	43.23 ± 7.12	0.19
BMD of lumbar spine(g/cm2)	0.71 ± 0.19	0.83 ± 0.27	0.22
Diagnosis(LSS/DDD)	60/14	81/33	0.17
Number of Inter-body cages(1/2)	60/14	96/18	0.69
Bone graft(autogenous/autogenous + allograft)	74/6	114/21	0.09
Symptomatic ASD	13	19	0.87

*BMI* Body mass index, *BMD* Bone mineral density

### Early R-ASD

**Fig. 2:**
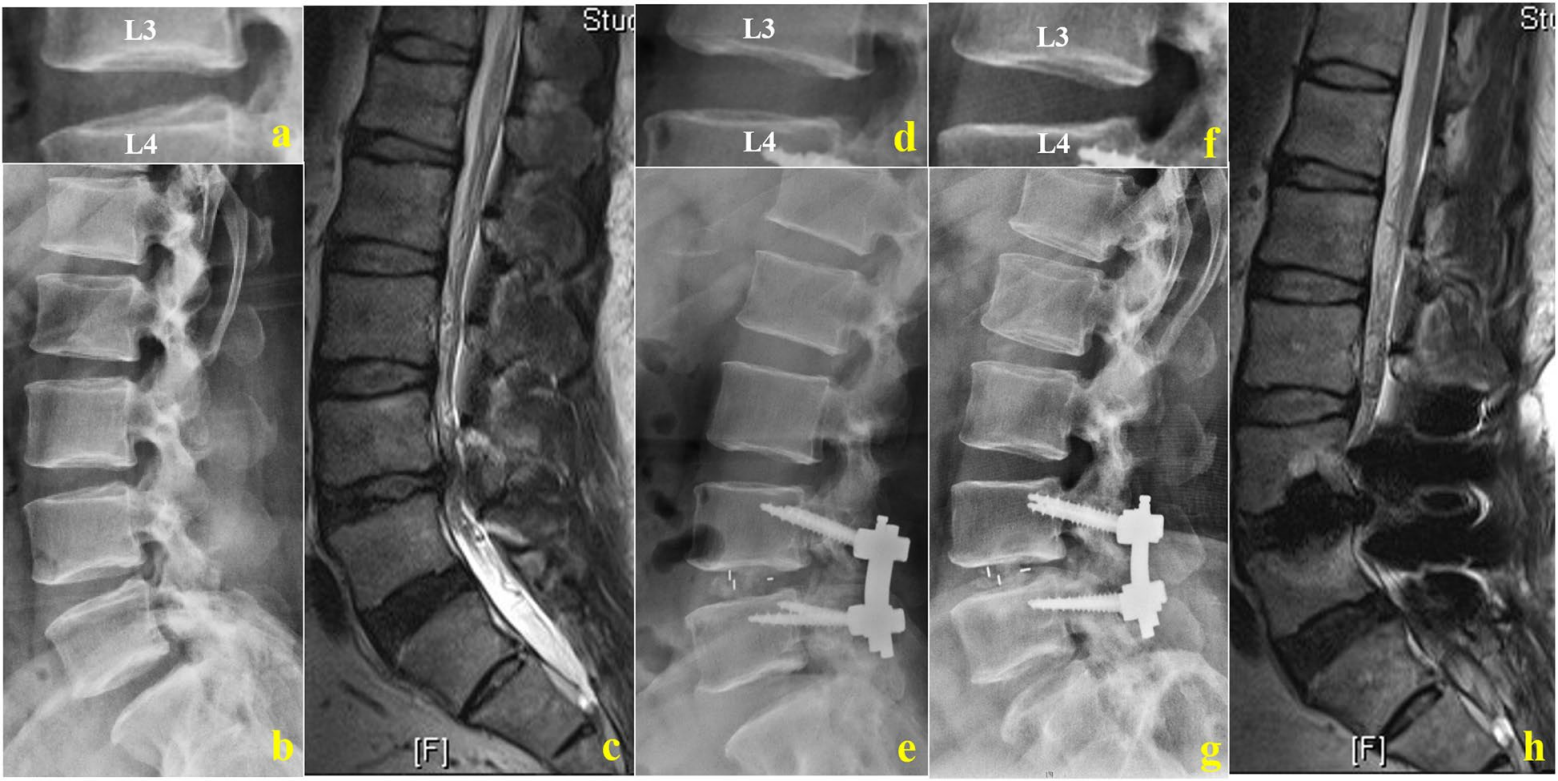
Radiological studies of a 75-year-old man with lumbar spinal stenosis after CBT-MIDLIF. **a**–**c** Preoperative plain radiographic image and preoperative sagittal magnetic resonance imaging (MRI) scan. Intervertebral space height (ISH): 14.67 mm, foraminal height (FH): 25.17 mm, foraminal width (FW): 9.98 mm, range of motion (ROM): 11.79°, lumbar lordosis (LL): 43.10°, sacral slope (SS): 36.60°, COBB 1: 8.30°, COBB 2: 18.32°, H 2/H 1: 3.18, and Pfirrmann classification of cephalic lumbar intervertebral disk: grade I. **d**, **e** Postoperative plain radiographic images at 6-month follow-up. **f**–**h** Postoperative plain radiographic image and preoperative sagittal MRI scan at 38-month follow-up. ISH: 13.60 mm, FH: 24.78 mm, FW: 10.79 mm, ROM: 12.92°, LL: 55.4°, SS: 44.70°, COBB 1: 9.10°, COBB 2: 19.15°, H 2/H 1: 3.06, and Pfirrmann classification: grade I

**Fig. 3:**
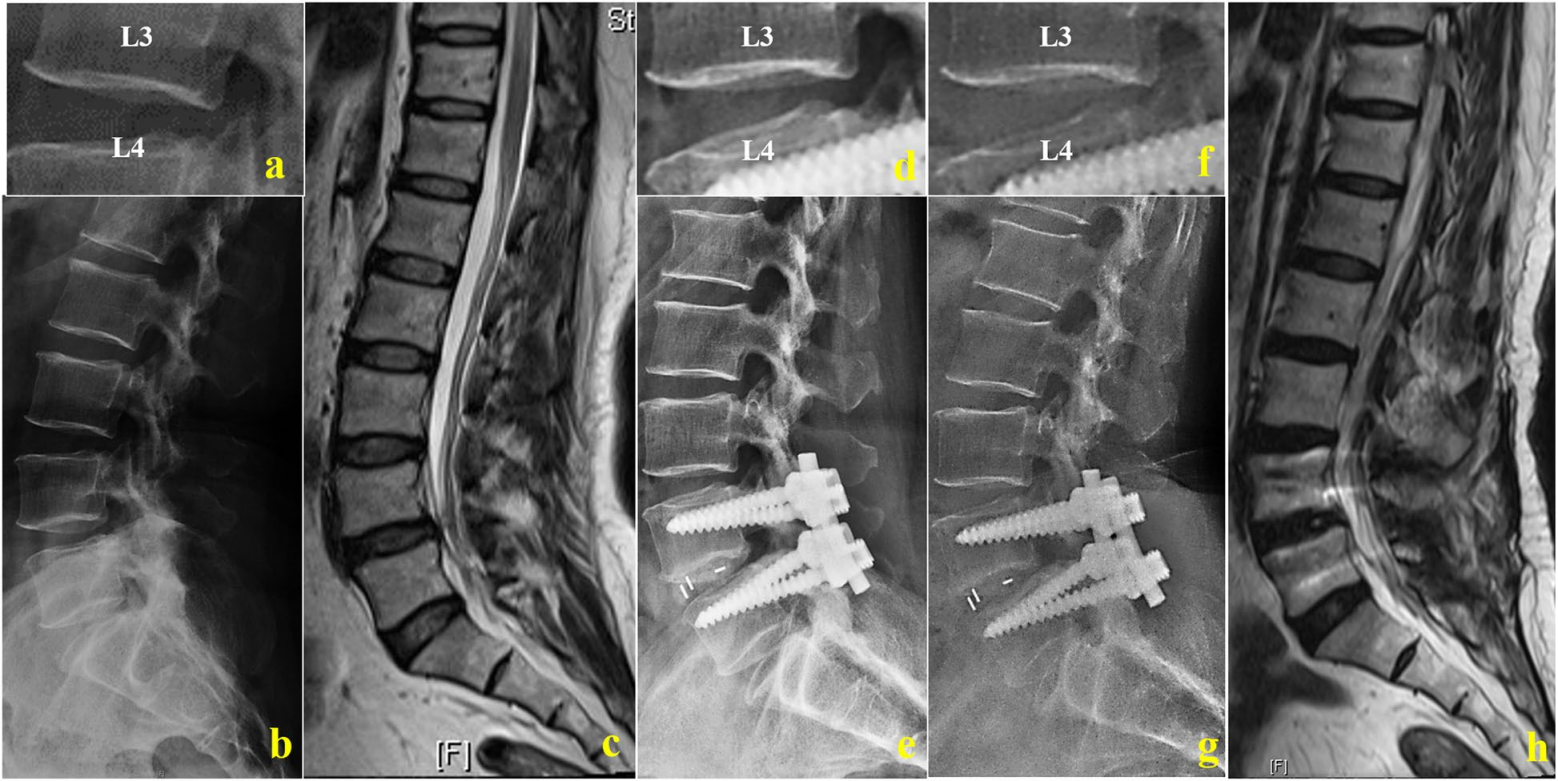
Radiological studies of a 76-year-old woman with lumbar spinal stenosis after PS-PLIF. **a**–**c** Preoperative plain radiographic image and preoperative sagittal magnetic resonance imaging (MRI) scan. Intervertebral space height (ISH): 13.03 mm, foraminal height (FH): 16.40 mm, foraminal width (FW): 9.12 mm, range of motion (ROM): 11.85°, lumbar lordosis (LL): 41.22°, sacral slope (SS): 31.95°, COBB 1: 13.90°, COBB 2: 21.40°, H 2/H 1: 2.61, and Pfirrmann classification of the cephalic lumbar intervertebral disk: grade I. **d**, **e** Postoperative plain radiographic images at 6-month follow-up. **f**–**h** Postoperative plain radiographic image and preoperative sagittal MRI scan at 38-month follow-up. ISH: 10.17 mm, FH: 14.66 mm, FW: 8.27 mm, ROM: 13.75°, LL: 56.12°, SS: 47.45°, COBB 1: 17.32°, COBB 2: 23.56°, H 2/H 1: 3.69, and Pfirrmann Classification: grade IV

The changes in the cephalad R-ASD parameters, including ISH, FH, FW, and ROM, are presented in Figs. 2 and 3, and Table 2. No significant changes in the 6-month postoperative cephalad R-ASD parameters were observed with CBT-MIDLIF (*p* > 0.05), while ISH, FH and FW were significantly less after PS-PLIF than before operation (*p* < 0.001). At the last follow-up, setting the preoperative values as the baseline, ISH, FH, and FW decreased from 11.52 ± 2.37 mm to 11.20 ± 1.82 mm (*p* < 0.001), from 20.85 ± 2.95 mm to 20.16 ± 2.43 mm (*p* < 0.001), and from 11.88 ± 2.73 mm to 11.40 ± 2.58 mm, respectively (*p* = 0.02). The ROM increased significantly from 11.63° ± 1.92° to 12.52° ± 2.01° (*p* = 0.03) with CBT-MIDLIF. While, ISH, FH, FW, and ROM showed significant changes with PS-PLIF in the long term after surgery, at the last follow-up (*p* < 0.001). Moreover, the changes in the cephalad ASD radiographic parameters in the patients who underwent PS-PLIF were more remarkable than those in the patients who underwent CBT-MIDLIF (*p* < 0.05), as described in Table 3.

**Table 2:** Cephalad adjacent segment degeneration–CBT Group VS. PS Group

Variable		Pre-operative	6 months FU		Last FU	
		**mean ± SD**	**mean ± SD**	***p* Value**	**mean ± SD**	***p* Value**
**CBT Group *N* = 74**	ISH (mm)	11.52 ± 2.37	11.44 ± 1.94	0.53	11.20 ± 1.82	0.00
	FH (mm)	20.85 ± 2.95	20.41 ± 2.57	0.24	20.16 ± 2.43	0.00
	FW (mm)	11.88 ± 2.73	11.91 ± 2.67	0.86	11.40 ± 2.58	0.02
	ROM (°)	11.63 ± 1.92	11.99 ± 1.62	0.15	12.52 ± 2.01	0.03
**PS Group *N* = 114**	ISH (mm)	12.08 ± 1.58	11.15 ± 1.79	0.00	10.60 ± 1.92	0.00
	FH (mm)	20.56 ± 4.17	19.35 ± 2.82	0.00	18.40 ± 2.81	0.00
	FW (mm)	12.42 ± 2.12	11.26 ± 1.69	0.00	10.11 ± 2.00	0.00
	ROM (°)	12.05 ± 2.21	12.93 ± 1.90	0.00	14.75 ± 1.53	0.00

**Table 3:** The change of cephalad adjacent segment degeneration

Variable	PS Group	CBT Group	*p* Value
	**mean** ± **SD**	**mean** ± **SD**	
6 months △ISH (mm)	0.94 ± 1.18	0.08 ± 1.11	0.00
6 months △FH (mm)	1.20 ± 2.95	0.45 ± 1.67	0.03
6 months △FW (mm)	1.16 ± 1.26	-0.03 ± 1.36	0.00
6 months △ROM (°)	0.88 ± 1.01	0.36 ± 0.89	0.12
Last FU △ISH (mm)	1.48 ± 0.90	0.32 ± 1.03	0.00
Last FU △FH (mm)	2.16 ± 2.80	0.69 ± 1.68	0.00
Last FU △FW (mm)	2.31 ± 1.60	0.48 ± 1.70	0.00
Last FU △ROM (°)	2.70 ± 1.87	0.89 ± 0.57	0.02

**Fig. 4:**
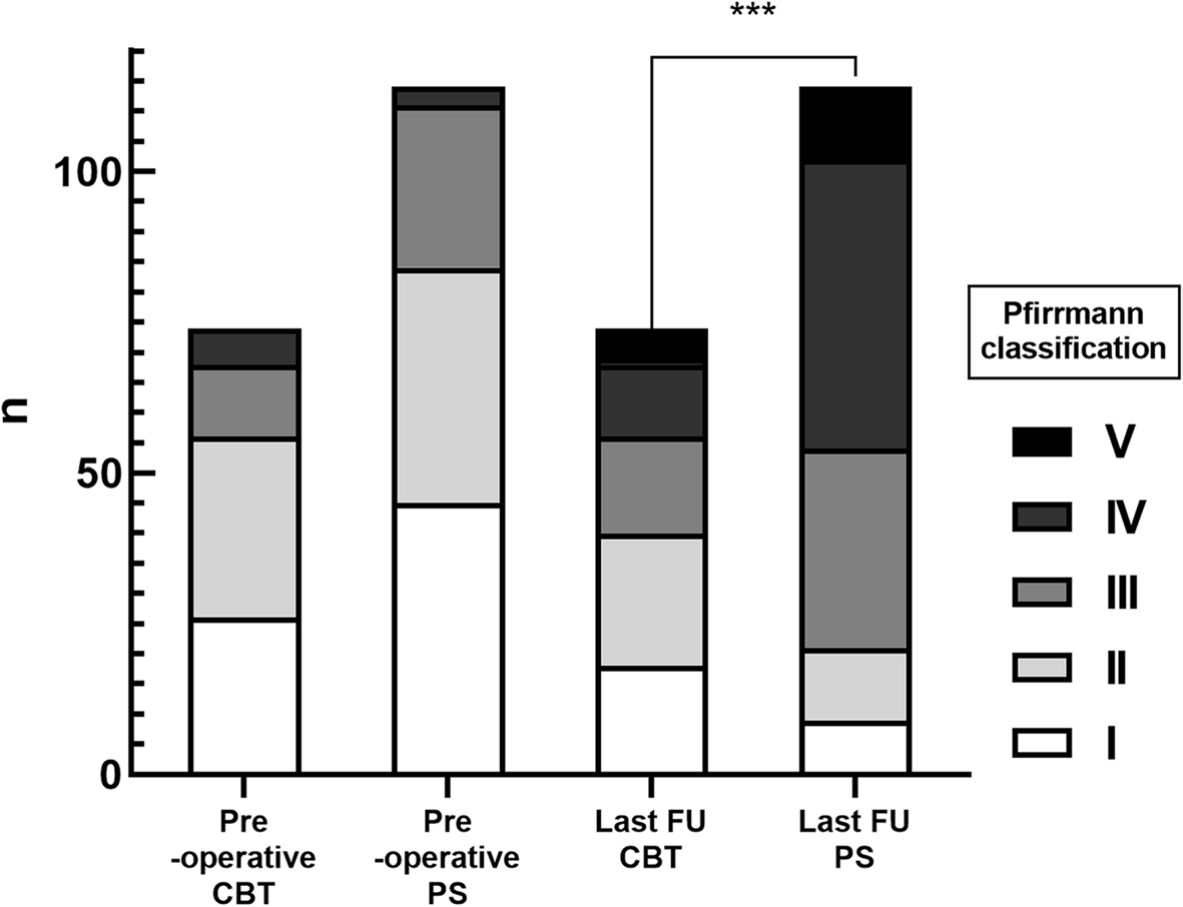
Adjacent segment degeneration of the cephalad spinal disk. A significant difference in cephalad spinal disk degeneration in the adjacent segment was observed with PS-PLIF as compared with CBT-MIDLIF (*p* < 0.001) at the last follow-up

The ASD of the L3/4 disc is shown in Fig. 4 and Table 4. No significant difference in the composition of disc grade was found between the two groups preoperatively (*p* > 0.05). Compared with the preoperative status, a significant degeneration of the L3/4 disc occurred in the adjacent segment with PS-PLIF at the last follow-up (45:39:27:3:0 vs. 9:12:33:48:12, *p* < 0.001). After CBT-MIDLIF, the number of degenerated L3/4 disc increased (26:30:12:6:0 vs. 18:22:16:12:6, *p* = 0.02). However, there was more significant degeneration of the cephalad spinal disc with PS-PLIF than CBT-MIDLIF at the last follow-up (*p* < 0.001).

**Table 4:** The adjacent segment degeneration of cephalad spinal disc (L3/4)

	PS Group	CBT Group		*p* Value
Pre-operative (I:II:III:IV:V)	45:39:27:3:0	26:30:12:6:0	Fisher	0.2
Last FU (I:II:III:IV:V)	9:12:33:48:12	18:22:16:12:6		0.02
		Fisher	/	/
*p* Value	0.00	0.02	/	/

I: the number of patients of Pfirrmann classification Grade I; II: the number of patients of Pfirrmann classification Grade II; III: the number of patients of Pfirrmann classification Grade III; IV: the number of patients of Pfirrmann classification Grade IV; V: the number of patients of Pfirrmann classification Grade V

### Regional lumbar sagittal alignment

Consistent with the larger SS (*p* < 0.001), LL was larger (*p* = 0.76) at 1-month follow-up than before operation, but not significantly after CBT-MIDLIF. Although SS showed the significant change (*p* < 0.001) after PS-PLIF instantaneously, LL increased from 39.05° ± 12.03° to 41.28° ± 12.38° (*p* < 0.001). At the last follow-up, the LL, SS, Cobb angle of the intervertebral space, L4–L5 Cobb angle, and height of the anterior edges of intervertebral space at L4–L5 were significantly greater than before operation in both groups (*p* < 0.001; Figs. 2 and 3, and Table 5). The differences between the lumbar sagittal alignment changes are shown in Table 6. The increases in LL, SS, L4–L5 Cobb and Cobb angle of the intervertebral space at L4–L5 were significantly less after CBT-MIDLIF than after PS-PLIF at the last follow-up (*p* < 0.001).

**Table 5:** Lumbar sagittal alignment–CBT Group VS. PS Group

Variable		Pre-operative	1 month FU		Last FU	
		**mean** ± **SD**	**mean** ± **SD**	***P* Value**	**mean** ± **SD**	***p* Value**
**CBT Group *N* = 74**	LL (°)	44.71 ± 8.97	44.88 ± 9.45	0.76	47.87 ± 9.00	0.02
	SS (°)	33.97 ± 7.36	38.33 ± 8.47	0.00	37.34 ± 8.27	0.00
	COBB 1 (°)	10.94 ± 3.79	13.15 ± 3.48	0.00	12.62 ± 3.34	0.00
	COBB 2 (°)	17.86 ± 5.95	19.70 ± 5.63	0.00	19.52 ± 5.54	0.00
	H 2 / H 1	2.32 ± 0.47	2.88 ± 0.70	0.00	3.06 ± 1.14	0.01
**PS Group *N* = 114**	LL (°)	39.05 ± 12.03	41.28 ± 12.38	0.00	47.48 ± 14.44	0.00
	SS (°)	31.81 ± 8.2	34.97 ± 7.39	0.00	38.19 ± 8.90	0.00
	COBB 1 (°)	6.53 ± 5.01	11.33 ± 3.12	0.00	11.73 ± 3.47	0.00
	COBB 2 (°)	13.58 ± 7.70	19.89 ± 5.31	0.00	20.43 ± 5.08	0.00
	H 2 / H 1	1.87 ± 0.87	2.21 ± 0.87	0.00	2.66 ± 0.90	0.00

Cobb 1: the Cobb angle of intervertebral space among L4-5; Cobb 2: L4-5 Cobb; H1: height of posterior edges of intervertebral space among L4-5; H2: height of anterior edges of intervertebral space among L4-5

**Table 6:** The change of Lumbar sagittal alignment

Variable	PS Group	CBT Group	*p* Value
	**mean** ± **SD**	**mean** ± **SD**	
1 month FU △LL (°)	2.22 ± 4.22	0.17 ± 5.02	0.03
1 month FU △SS (°)	3.15 ± 7.42	4.36 ± 5.54	0.23
1 month FU △COBB 1 (°)	4.80 ± 3.20	2.21 ± 1.34	0.00
1 month FU △COBB 2 (°)	6.31 ± 4.47	1.84 ± 1.28	0.00
1 month FU △H 2 / H 1	0.34 ± 0.97	0.56 ± 0.47	0.06
Last FU △LL (°)	8.42 ± 8.02	3.16 ± 7.20	0.00
Last FU △SS (°)	6.38 ± 8.58	3.38 ± 5.69	0.00
Last FU △COBB 1 (°)	5.20 ± 3.27	1.69 ± 1.79	0.00
Last FU △COBB 2 (°)	6.85 ± 4.58	1.66 ± 1.11	0.00
Last FU △H 2 / H 1	0.79 ± 0.71	0.69 ± 0.98	0.50

Cobb 1: the Cobb angle of intervertebral space among L4-5; Cobb 2: L4-5 Cobb; H1: height of posterior edges of intervertebral space among L4-5; H2: height of anterior edges of intervertebral space among L4-5.

### Clinical outcome

**Fig. 5:**
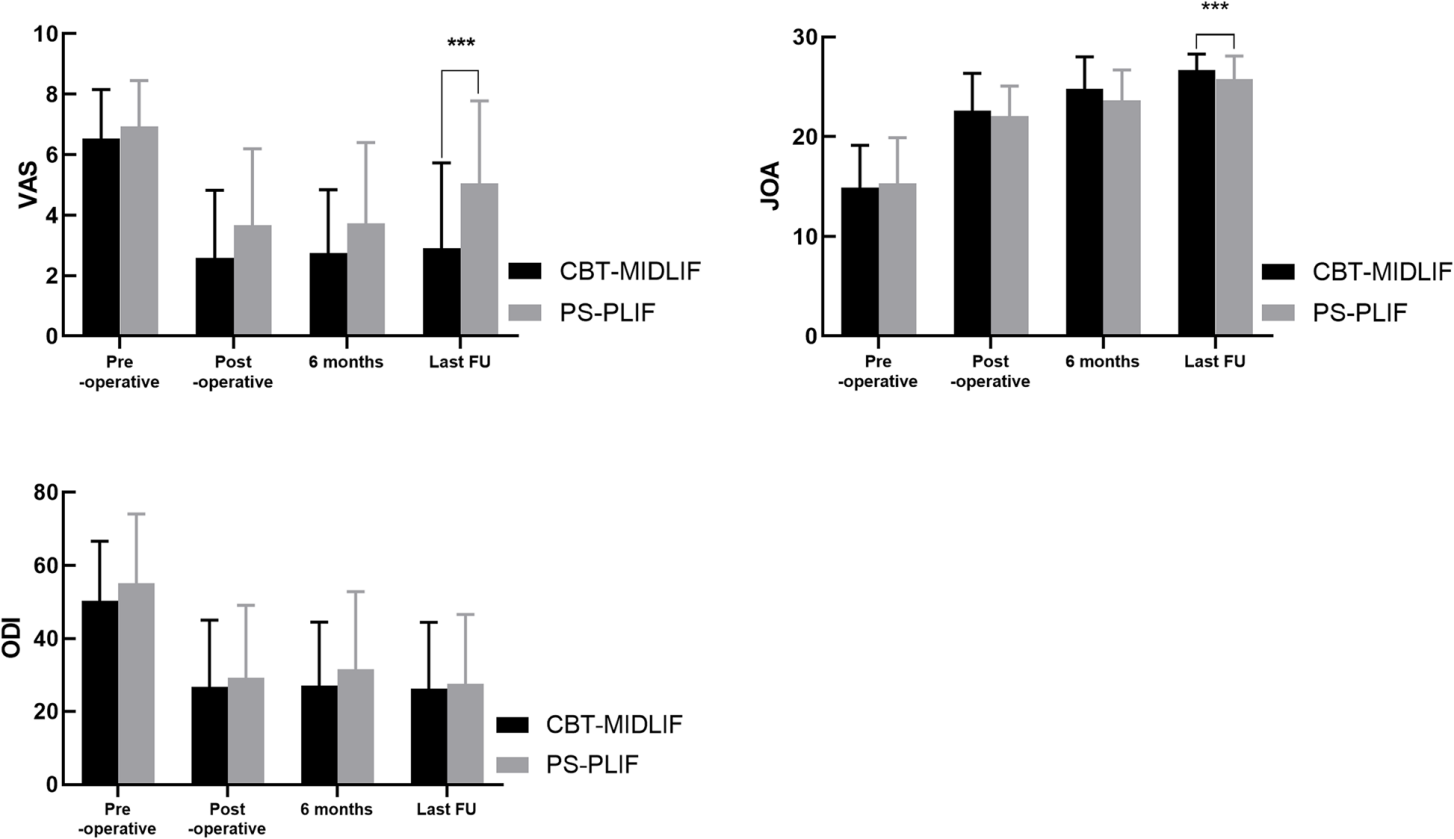
Clinical outcomes in the traditional pedicle screw and cortical bone trajectory groups. The symptom caused by degenerative spinal disorders was significantly improved after transforaminal lumbar interbody fusion surgery in both groups (*p* < 0.01). Significant differences in visual analog scale and Japanese Orthopedic Association (JOA) scores at the last follow-up were found between the two groups (*p* < 0.01)

The clinical outcomes before operation and at the 1-month, 6-month, and last follow-up are shown in Fig. 5. No significant differences were found between the two groups in preoperative VAS (6.53 vs. 7.92), ODI (50.31 vs. 55.14), and JOA scores (14.84 vs. 15.30; *p* > 0.05). Compared with the preoperative values, all clinical outcomes, including VAS, ODI, and JOA scores, significantly improved after the CBT-MIDLIF or PS-PLIF (*p* < 0.01). At the last follow-up, the clinical parameters favored CBT-MIDLIF, with less back and leg pains according to the lower VAS score (2.90 vs. 5.05, *p* < 0.001) and JOA scores (26.72 vs. 25.77, *p* = 0.03) than those after PS-PLIF. However, the ODI score showed no significant difference between the two groups postoperatively (26.73 vs. 29.32, *p* = 0.11) and at the last follow-up (26.60 vs. 27.55, *p* = 0.09).

## Discussion

With a minimum 3-year follow-up of 188 patients, more attention should be paid to the inherent advantages and disadvantages of CBT-MIDLIF and PS-PLIF in terms of early R-ASD and regional lumbar sagittal alignment changes when developing surgical plans and strategies. Not only the bone structure but also the cephalad spinal disc showed less degeneration in the adjacent segments with CBT-MIDLIF than with PS-PLIF. Nevertheless, PS-PLIF was regarded to have a stronger compression ability at L4/L5. Although CBT-MIDLIF had a slight advantage in terms of long-term outcome, the surgery outcomes were satisfactory both after CBT-MIDLIF and PS-PLIF.

The incidence of ASD associated with arthrodesis was 34%, which is one of the common complications after lumbar interbody fusion [12]. More than two to five years after surgery, Xia et al. [13] reported that the incidence of R-ASD (33.6%) was higher than that of S-ASD (12.1%). We can better follow up and observe the adjacent segmental degeneration of patients by cephalad R-ASD parameters. Therefore, we adopted the cephalad R-ASD parameters that can better react to changes at L3–L4 to observe R-ASD rather than S-ASD at an early stage. We found the more significant change after PS-PLIF than CBT-MIDLIF at the last follow-up. This was similar to the tendency proved by Sakaura et al. [14] that PLIF with CBT screw fixation significantly reduced the incidence of early cephalad R-ASD as compared with PS-PLIF (*p* < 0.05). Wang et al. [15] found that intraoperative superior facet joint violation was a risk factor of ASD. With its difference from PS-PLIF, CBT-MIDLIF caused less destruction of the superior facet joint and paravertebral muscles, [8] which is sufficient to explain the reasons for our results.

We also found a significant difference in cephalad spinal disc degeneration in the cephalic segment with PS-PLIF as compared with CBT-MIDLIF (*p* = 0.02) at the last follow-up. Increased facet loads and adjacent level motions accelerate disc degeneration [16]. Moreover, by performing a finite element analysis, Kim et al. [17] demonstrated that intraoperative superior facet joint violation increases the disc stresses and facet contact forces, and then accelerate the degeneration of the joint and adjacent disc segment, which leads to ASD. Higher BMI, age > 60 years, preoperative disc degeneration at the adjacent segment, excessive disc height distraction, and intraoperative superior facet joint violation were considered as the other risk factors of ASD [18–20]. The correction of disc height seems to be more in PS group and more amount of correction in PS-PLIF group added more stress to cranial fact joint. Biomechanical experiments need to be carried out to verify the difference of correction stress to facet joint. Although not all radiographic changes correlate with the patient’s clinical symptoms, [21] CBT screw fixation is a good option for patients with the above-mentioned risk factors when surgical planning and strategies are developed.

Moreover, inadequate restoration of lordosis at L4 to S1 is relevant to ASD [22]. A significant improvement in lumbar sagittal alignment was observed after the operations in both groups (*p* < 0.001) in our study. The LL increased respectively in 3.165° ± 7.20° and 8.42° ± 8.02° with CBT-MIDLIF and PS-PLIF, respectively (*p* < 0.001). Carlson et al. [23] reported that LL could be restored to the same level of 45.00° ± 7.40° after the operations. Another study demonstrated that the maintenance of lordotic angles with CBT pedicle screw placement was similar to the conventional or percutaneous methods, which correspond with Kasukawa’s study [24]. Although immediate difference in LL was found after PS-PLIF but not after CBT-MIDLIF, we could not emphasize the accurate clinical significance. Uribe et al. [25] found that patients with hypo lordosis had a more considerable LL postoperatively than those with normal lordosis, whereas we found a lower preoperative LL in the CBT-MIDLIF group than in the PS-PLIF group in our study. Furthermore, L4/L5 PS-PLIF may have a stronger compression ability because of the shorter screw length, smaller pathway depth, and shorter distance of the moment [26].

The clinical outcome after CBT screw placement has been reported extensively, but most studies were limited to a single evaluation tool and short follow-up time. VAS and JOA scores were used to assess the degree of low back and leg pains, and the ODI was measured to evaluate the lumbar disability in our study with > 3 years of follow-up. At the last follow-up, significant differences were observed in the VAS and JOA scores (*p* < 0.01) between the two groups, which is similar to the findings of Sakaura et al. [14]. At the same time, these findings well explain that the differences in clinical outcome corresponded to the changes in ASD. To some extent, this was related to the protection of the superior facet joints and muscles, the path parallel to the cephalad endplate, and the minimum damage to the approach of the CBT. However, no significant difference in ODI was found between CBT-MIDLIF and PS-PLIF at all time points, which is inconsistent with the report of Crawford et al. [27] because of the differences in follow-up time and functional effects of ASD.

Since the development of the CBT screw technique in our institution in 2015, > 450 patients have undergone CBT-MIDLIF. More can be accomplished during the operation to reduce the incidence of postoperative ASD and restore normal LL sagittal alignment according to clinical experience and attempt CBT-MIDLIF. We recommend starting with a more caudal midline incision, referring to fluoroscopy to protect against the cephalic soft tissue and tension band disruption posteriorly. The paraspinal muscle must be exposed meticulously and kept from the casual traction of the paraspinal muscles. The risk of intraoperative superior facet joint violation could be minimized by adopting the intersection technique and lateral-cephalic CBT rigidly described in the surgical technique. Meanwhile, this could help prevent screw misplacement. However, if the proximal length of the rod is too long, a collision in the upper facet will occur, accelerating the occurrence of ASD. As the particularity of CBT, the surgeon must pay attention to the rod length during rod placement.

One limitation of our study is that the 3-year follow-up is still relatively short considering that CBT as a new technique has only been used in recent years, and the apparent improvement of symptoms after surgery is the reason for the difficulty of follow-up. As a retrospective cohort study, the PS-PLIF cohort and CBT-MIDLIF cohort included in the retrospective cases in our study had selection bias due to patients’ and surgeons’ selection. To better detective the postoperative changes at L4–L5 after surgery using different instruments, the patients were strictly included on the basis of the criteria, which also made the sample size relatively small. Therefore, it requires us that randomized controlled trials must be performed in a multicenter to obtain more convincing conclusions and a biomechanical experiment must be performed to explain the associated mechanism.

## Conclusions

CBT-MIDLIF had less radiographic degeneration in the adjacent segment than PS-PLIF at 3-year follow-up. The lumbar sagittal alignment could be improved significantly and the surgical outcomes were satisfactory after either CBT-MIDLIF or PS-PLIF.

## Data Availability

The datasets generated and analysed during the current study are not publicly available due official requirements for the author's doctoral dissertation in writing, but are available from the corresponding author on reasonable request with ease.

## Abbreviations

ASD: Adjacent segment degeneration
R-ASD: Radiographic adjacent segment degeneration
MIDLIF: Midline lumbar interbody fusion
CBT: Cortical bone trajectory
PS: Pedicle screw
PLIF: Posterior lumbar interbody fusion
ISH: Intervertebral space height
FH: Foraminal height
FW: Foraminal width
LL: Lumbar lordosis
SS: Sacral slope
ROM: Range of motion
ODI: Oswestry disability index
VAS: Visual analog scale
JOA: Japanese Orthopedic Association
